# The cutting-edge morphology of the mole snake’s dental apparatus

**DOI:** 10.7717/peerj.6943

**Published:** 2019-06-07

**Authors:** Alexandra M. Evans, Jonah N. Choiniere, Graham J. Alexander

**Affiliations:** 1 School of Animal, Plant and Environmental Sciences, University of the Witwatersrand, Johannesburg, South Africa; 2 Evolutionary Studies Institute, University of the Witwatersrand, Johannesburg, South Africa; 3 School of Geosciences, University of the Witwatersrand, Johannesburg, South Africa

**Keywords:** Mole snake, Dentition, Sexual dimorphism, Functional morphology

## Abstract

The mole snake (*Pseudaspis cana*) is capable of inflicting unusual bites in defence and during male combat that present as two parallel lacerations. We investigated the dental morphology of the mole snake by making SEM images, and by CT-scanning and digitally reconstructing the skulls of 14 specimens comprising both sexes. The lengths, volumes, shapes and positions of maxillary and dentary teeth were compared within individuals, between individuals, and between sexes. CT reconstructions show the occurrence of large, flat triangular teeth at the posterior end of the maxilla that are angled to point towards the posterior of the skull. SEM imagery highlights the presence of sharp ridges (carinae) on the posterior edges of the posterior dentary and maxillary teeth. Males have greater dental specialization, maxillary tooth variation, enlargement of the posterior-most maxillary teeth, and dentary teeth with posterior carinae. We hypothesize that mole snake dental specializations are adaptations for their particular form of male combat and possibly for subduing prey in the confines of underground burrows. Our findings reveal a complex dental morphology in mole snakes and provide impetus for further studies on the functional morphology of snake teeth.

## Introduction

Snakes are highly successful predators that have colonised numerous habitats (Gans, 1961; Greene, 1983). Their success is partly due to a specialised morphology, characteristically a long body, no functional limbs, a highly kinetic cranium, and distinctive teeth (Greene, 1997; Cundall & Greene, 2000; Longrich, Bhullar & Gauthier, 2012). The morphology of snake jaws is adapted to accommodate the swallowing of prey whole, with cranial kinesis and unfused dentary bones (mandibular symphysis) allowing for a wide gape (Cundall & Greene, 2000), and there is a diverse range of tooth shapes and arrangements to aid in prey capture (Greene, 1997; Knox & Jackson, 2010; Vaeth, Rossman & Shoop, 1985). However, the diversity of snake dentition has been sparsely researched in recent years, especially for non-venomous species, despite its importance in snake ecology and its interesting variability across taxa.

Studies of the dental morphology of snakes have primarily focused on iconic venom-delivery systems (e.g.,  Fry et al., 2012; Jackson, 2003; Vaeth, Rossman & Shoop, 1985). A snake fang is generally described as a long, pointed maxillary tooth modified to carry and dispense venom into the tissue of prey or attacker (Jackson, 2003). Fangs may be tubular, grooved, or ungrooved, with ungrooved being differentiated from other enlarged teeth by the presence of ridges along the distal rostral and caudal surface of the tooth and the presence of a venom gland (Jackson & Fritts, 1995; Jackson, 2007). Venom may have evolved to aid in prey capture and defence (Fry et al., 2006; Casewell et al., 2013) and possibly played a role in facilitating digestion (Savitzky, 1980; Thomas & Pough, 1979), but see McCue (2007) for an alternate view. Despite the absence of tubular or grooved structures in regular teeth, the dentition of non-venomous snake species is not necessarily simple. Dental ridges are fairly common in both the dentary and maxillary teeth of colubrids such as the Dipsadinae and the Natricinae, as well as the aquatic Homalopsidae, all of which have diverse diets and habitats (Bogert, 1964; Vaeth, Rossman & Shoop, 1985; Young & Kardong, 1996).

The modification of snake teeth likely contributed to the evolution and radiation of snakes (Jackson, 2003; De Oliveira et al., 2016; Savitzky, 1980) due to the new feeding opportunities involving prey shape and size that this offered (Gans, 1961; Greene, 1983). In colubrids, diet (rather than phylogenetic history) appears to act as the greatest selective pressure for the evolution of specialised maxillary dentition in particular (Knox & Jackson, 2010). Britt, Clark & Bennett (2009) postulate that dental morphology in garter snakes is directly related to dietary preference and that specialist feeders tend to have dental features such as ridges that are absent in closely related generalist feeders. The maxillary teeth of the North American western terrestrial garter snake, *Thamnophis elegans,* and of the Southeast Asian common wolf snake, *Lycodon aulicus capucinus,* have sharp ridges, or carinae, on their posterior edges (Jackson & Fritts, 2004; Wright, Kardong & Bentley, 1979). This may be an adaptation to cutting through skink scales, promoting deep tooth penetration into the prey, and preventing the prey from escaping the mouth whilst being eaten. The teeth of the North American redbelly snake, *Storeria occipitomaculata*, also have distal carinae which aid in securing slippery gastropod prey (Do Amaral, 1999). Thus the dental morphology of non-venomous snakes is strongly linked to prey type.

The mole snake (*Pseudaspis cana,* Linnaeus 1758) is a non-venomous lamprophiid which, when handled, is capable of inflicting painful bites sometimes requiring suturing, the wounds being more severe than is usual for non-venomous snake bites. The structure of the teeth responsible for these bites has not previously been described, but they are hypothesised to aid in prey handling in confined spaces, defence from mole rats, or male combat (Alexander & Marais, 2007; Broadley, 1983). Mole snakes include moles and fossorial rodents in their diet (Alexander & Marais, 2007), and it is possible that the difficulty of hunting in restricted burrows could have driven the evolution of the development of this biting behaviour and corresponding dental morphology (Branch, 1998). Juvenile mole snakes have been recorded preying on skinks (Broadley, 1983), and thus another possible function of the sharp teeth is to aid in cutting through skink scales (Branch, 1998). The western keeled snake, *Pythonodipsas carinata*, the sister taxon to *Pseudaspis*, also has specialised dental morphology—it is believed that its enlarged palatine fangs aid in extracting prey from burrows (Branch et al., 1997). In a similar way, diet and hunting environment may have driven the development of the mole snake’s unusual bite.

Evidence of the mole snake’s distinctive bite has also been found on conspecific males as a result of male-male combat (G Alexander, pers. obs., 2015). Male-male combat has been described in many snake species and characteristically involves ritualised wrestling (Shaw, 1951; Carpenter, 1977; Blouin-Demers, Gibbs & Weatherhead, 2005). Mole snakes are unusual in the respect that male-male combat includes bites which can result in injury (Greene, 1997; Branch, 1998) and the wounds inflicted by male mole snakes on rivals present as paired parallel cuts across and around the body (G Alexander & A Evans, pers. obs., 2015, Figs. 1A & 1B).

**Figure 1:**
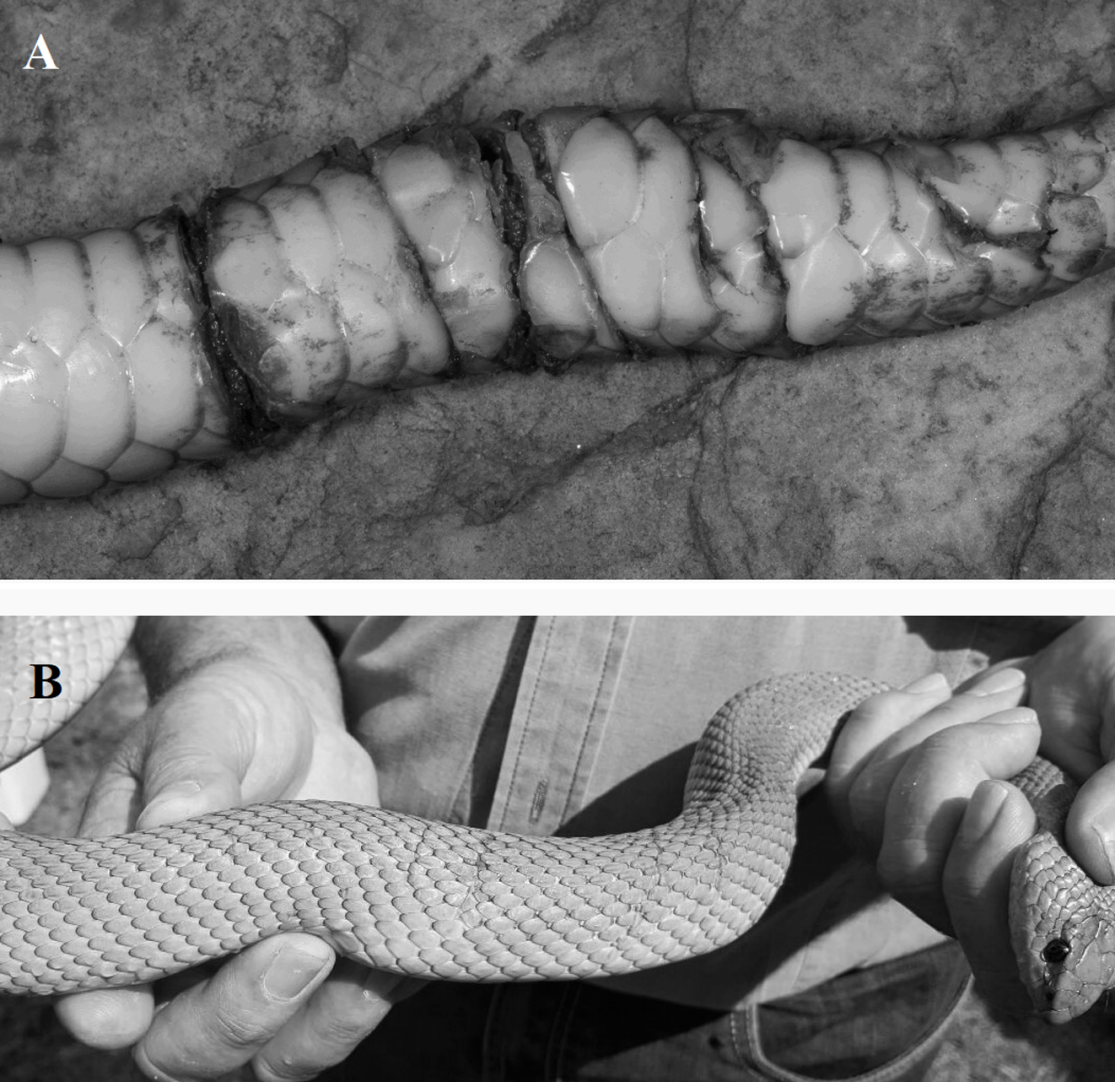
Photographs of fresh wounds (A) and scars of past wounds (B) on male mole snakes, sustained during male-male combat. Photo credit: Graham Alexander (A), Nicholas Evans (B).

We studied the external structure of mole snake teeth using micro-CT (computed tomography) scanning and SEM (scanning electron microscopy) imaging to investigate the teeth responsible for inflicting the characteristic parallel cuts. We also investigated whether sexual dimorphism in tooth size, variation and shape is present in mole snake dentition, and in the incidence of bite wounds on museum specimens. If mole snakes have heterogeneous dentition along the tooth row and show significant differences in tooth shape and size between sexes, then it is likely that males are employing these teeth for a combat success strategy, especially if the parallel bite marks are only present on male specimens. Features that appear in both males and females are likely more indicative of prey specialization or another, non-combat related adaptation.

## Materials & Methods

The heads of 14 *Pseudaspis cana* (Linnaeus 1758) (7 ♂; 7 ♀) specimens collected in Gauteng Province, South Africa, were scanned using micro computed tomography (micro-CT) at the Microfocus X-ray CT Facility at the Evolutionary Sciences Institute at the University of the Witwatersrand, Johannesburg (the use of the specimens was approved by the Animal Research Ethics Committee, University of the Witwatersrand, waiver no. 2004/28/1). The sex of each individual was assessed by cloacal probing. Eight specimens were found freshly killed (road kill) and the remaining specimens (preserved in ethanol or formalin) were loaned from the Ditsong Museum of Natural History, Gauteng, South Africa. Larger specimens (head length >40 mm in length) were scanned at 70 kV, 120 µA, 1 fps, 1 fa and 2,000 projections, and smaller specimens (head length <40 mm in length) at 120 kV, 150 µA, 1 fps, 1 fa and 2,000 projections. The CT scans of the crania and dental apparatus of each specimen were reconstructed with CT Pro 3D and then digitally segmented and analysed using the CT visualization software VG Studio Max 3.0 (Volume Graphics, 2014). This software allowed for the building of precise, high-resolution 3D models of the snake’s dental apparatus and its connection to the cranial skeleton.

Dentary teeth were investigated by counting the number of teeth with sharp edges and comparing between individuals and between sexes. The noticeable variation in the size of maxillary teeth at different positions along the jaw (unlike the fairly uniform size of the dentary teeth) motivated us to measure and compare the volumes of the maxillary teeth. Maxillary teeth were analysed by comparing position, shape and size of teeth at different positions on the maxilla for each individual. Volumes were extracted in VG Studio Max 3.0 after digitally segmenting individual teeth and the jaws from the skull. Tooth lengths were measured using Avizo 9 but were not used in analysis because the variation in degree of tooth curvature (which was not measured due to the high variation within and between individuals) meant that the lengths did not give a reliable measure of tooth size.

**Figure 2:**
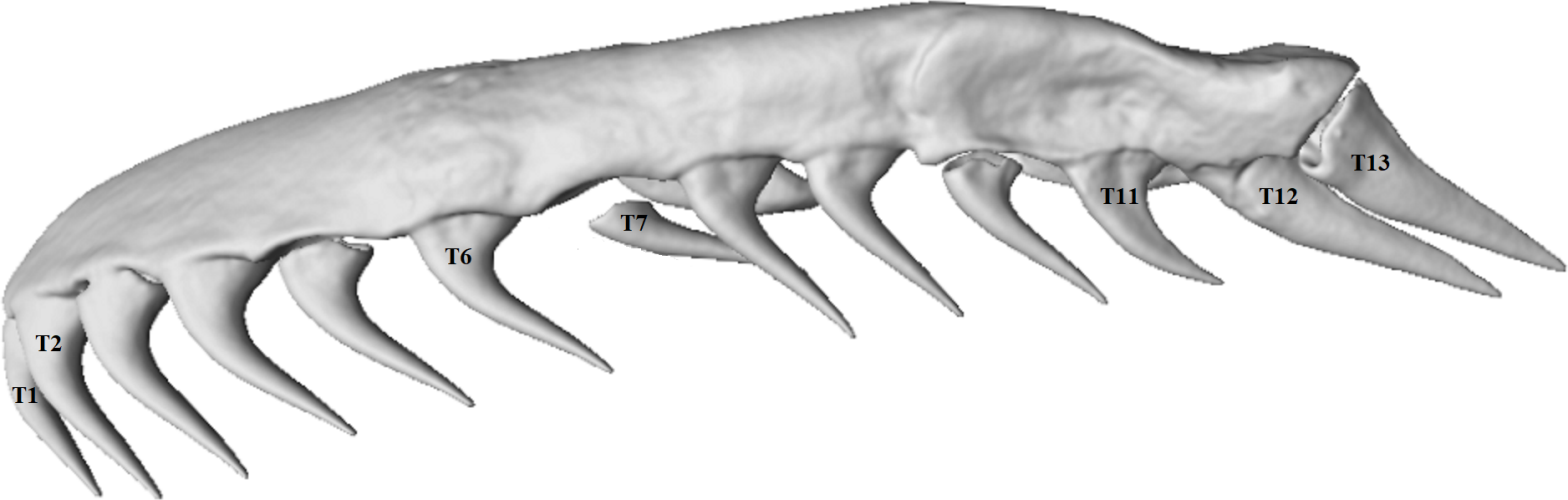
Micro-CT (computed tomography) reconstruction of the left maxilla of a mole snake, showing the maxillary teeth chosen for statistical analyses.

Maxillary tooth measurements were taken for the two most anterior teeth (T1 and T2), two teeth midway on the maxilla (T6 and T7) and the three most posterior teeth (T11–T13) of both left and right maxillae in order to investigate tooth variation along the maxilla (Fig. 2). The values for under-developed teeth (not yet attached to the dentigerous element) were removed from analysis. Tooth volume was measured as the total volume of the digitally-segmented tooth. The volumes of the lower right and left jaws (the compound and dentary bones together) were measured and used as a proxy for skull size and a means of estimating the effects of allometry because of the lower jaw’s fairly rigid structure. The joint between the dentary and compound bones is filled with collagenous tissue that may allow a small amount of movement, but this is very limited in comparison to the movement, and thus the additive error, that would be involved in measuring an entire skull with multiple kinetic parts.

The incidence of mole snake bites was investigated in 50 specimens from the Ditsong Museum of Natural History. Each snake was examined for the evidence of open wounds or scars, and the sex was noted. We recorded all cuts across the body and noted the severity. Although it is not possible to always conclusively distinguish wounds made from conspecific combat from those resulting from other causes, it is assumed that transverse cuts of >25% of the body width, in particular the spiral cuts, were likely inflicted in male-male combat, while grazed scales or very short scars (<25% of the width of the dorsal surface) were assumed not to be the result of conspecific bites. The positions of cuts were recorded as either in the tail region (from the cloaca to the tip of the tail) or in the torso region of each specimen.

Tooth detail was investigated qualitatively with scanning electron microscopy (SEM). Teeth of interest (by examination of CT 3D models) were coated with 10 nm carbon and a 5 nm gold-palladium (60:40) alloy, and were then micrographed using the FEI Quanta 200 E-SEM at the Microscopy and Microanalysis Unit in the school of Animal, Plant and Environmental Sciences at the University of the Witwatersrand, Johannesburg.

### Data analyses

All statistical analyses were performed using RStudio version 3.2.5 (R Core Team, 2014). The dental volume measurements were used in a series of comparisons, tooth by tooth, within individuals, between individuals, and between the sexes. To account for differences in individual snake size, the tooth volumes of each individual were first divided by the lower jaw volume of the respective individual. A mean value of left and right teeth for each of the teeth being measured was calculated for each specimen (e.g., volumes of T1 on the left maxilla and T1 on the right maxilla to give a mean T1 volume for the specimen). The same was done for the lower left and right jaw measurements, to give a mean lower jaw volume.

After testing for normality using a Shapiro–Wilk test, one-way ANOVAs were used to measure within-individual variation in log-transformed volumes of teeth at different positions along the maxilla. Sex differences were tested using a multifactoral ANOVA with sex as a factor. Tests were performed with seven mean maxillary tooth volumes at the positions described above (T1, T2, T6, T7, T11, T12 and T13; see Fig. 2). Wilcoxon rank-sum tests were used to test whether the volumes of the lower jaws, number of dentary teeth and mean volumes of maxillary teeth differed between male and female mole snakes. A chi-square test tested whether there was an association between incidence of conspecific bite wounds and sex.

## Results

### Maxillary and dentary tooth morphology of the mole snake

**Figure 3:**
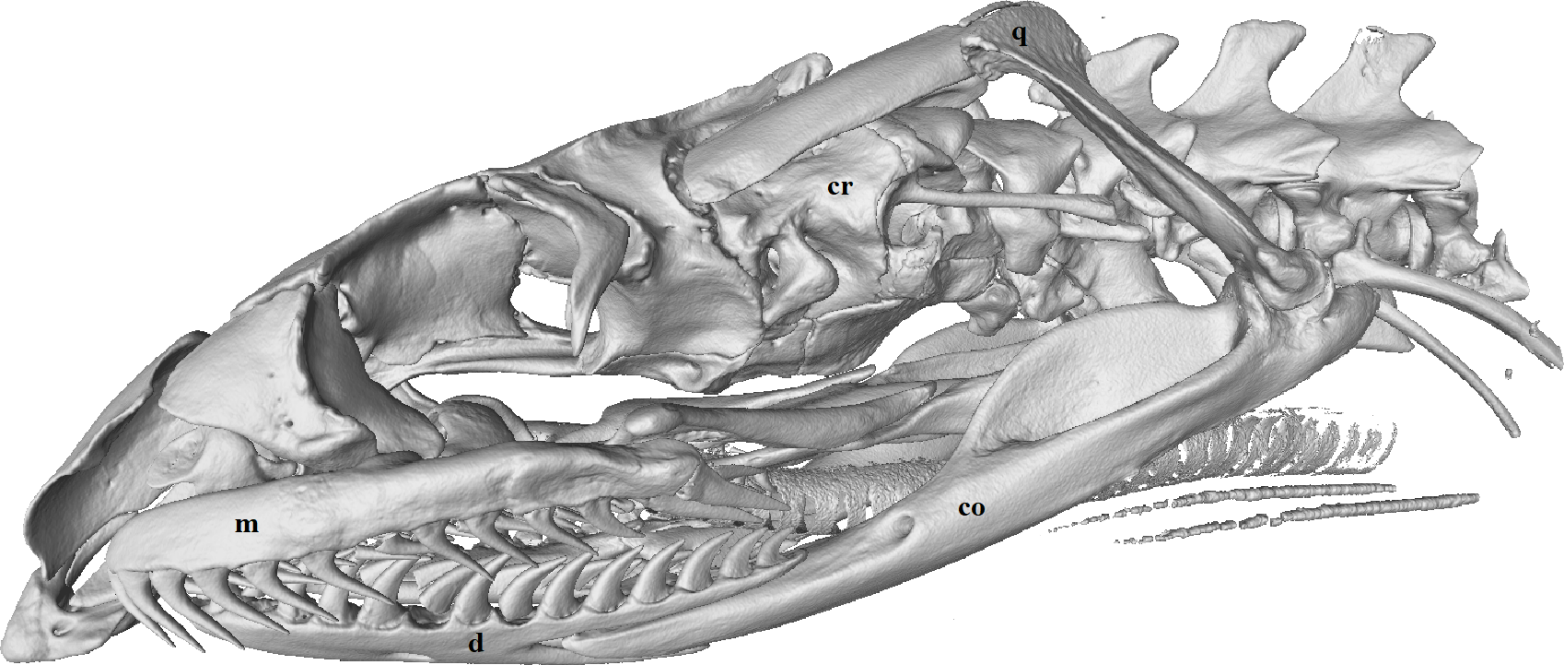
Micro-CT (computed tomography) reconstruction of the left lateral view of a male mole snake skull showing the cranium (cr), quadrate (q), compound (co), dentary (d) and maxilla (m) bones.

Each maxilla and dentary bone had a continuous row of solid acrodont teeth (attached directly onto the jaw bone) which varied in size and shape (Figs. 2 and 3). Replacement teeth (removed from CT images) lay to the medial side of the functional teeth.

### Dentary teeth

**Figure 4:**
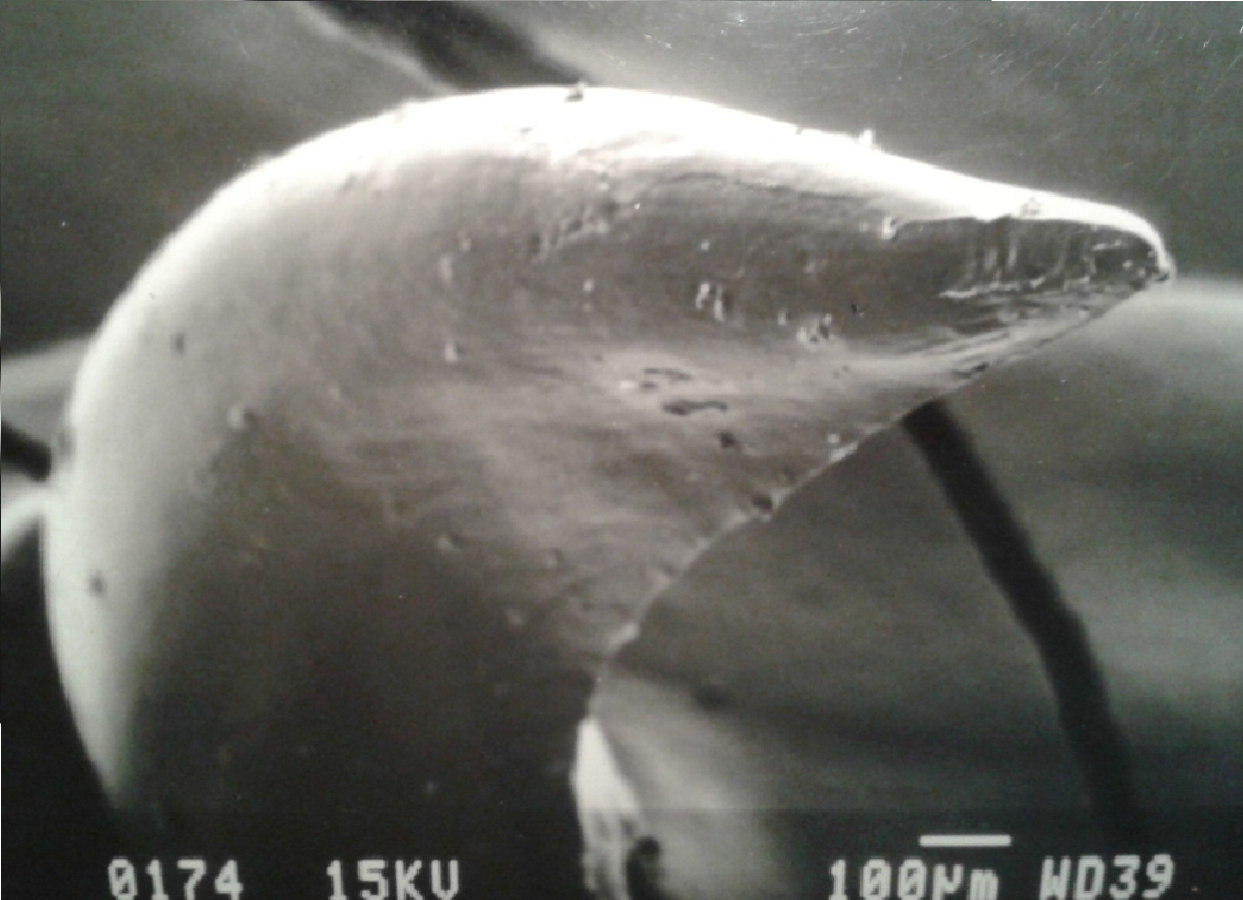
SEM (Scanning-Electron Microscopy) image of a posterior dentary tooth of a male mole snake, highlighting the blade-like carina on the posterior edge.

Individual dentary bones each had between 14 and 16 teeth. These teeth were recurved posteriorly, and some had posterior carinae (sharp ridges/edges) (Figs. 3 and 4), while the teeth without sharp edges were simply conical, tapering to a point. The number of teeth with sharp carinae varied between individual snakes, ranging from 5 to 13 on each dentary bone, and were absent in some specimens (see *Sex-based differences in relative tooth shape and volume* below). When present, carinae occurred in the posterior-most teeth.

### Maxillary teeth

**Figure 5:**
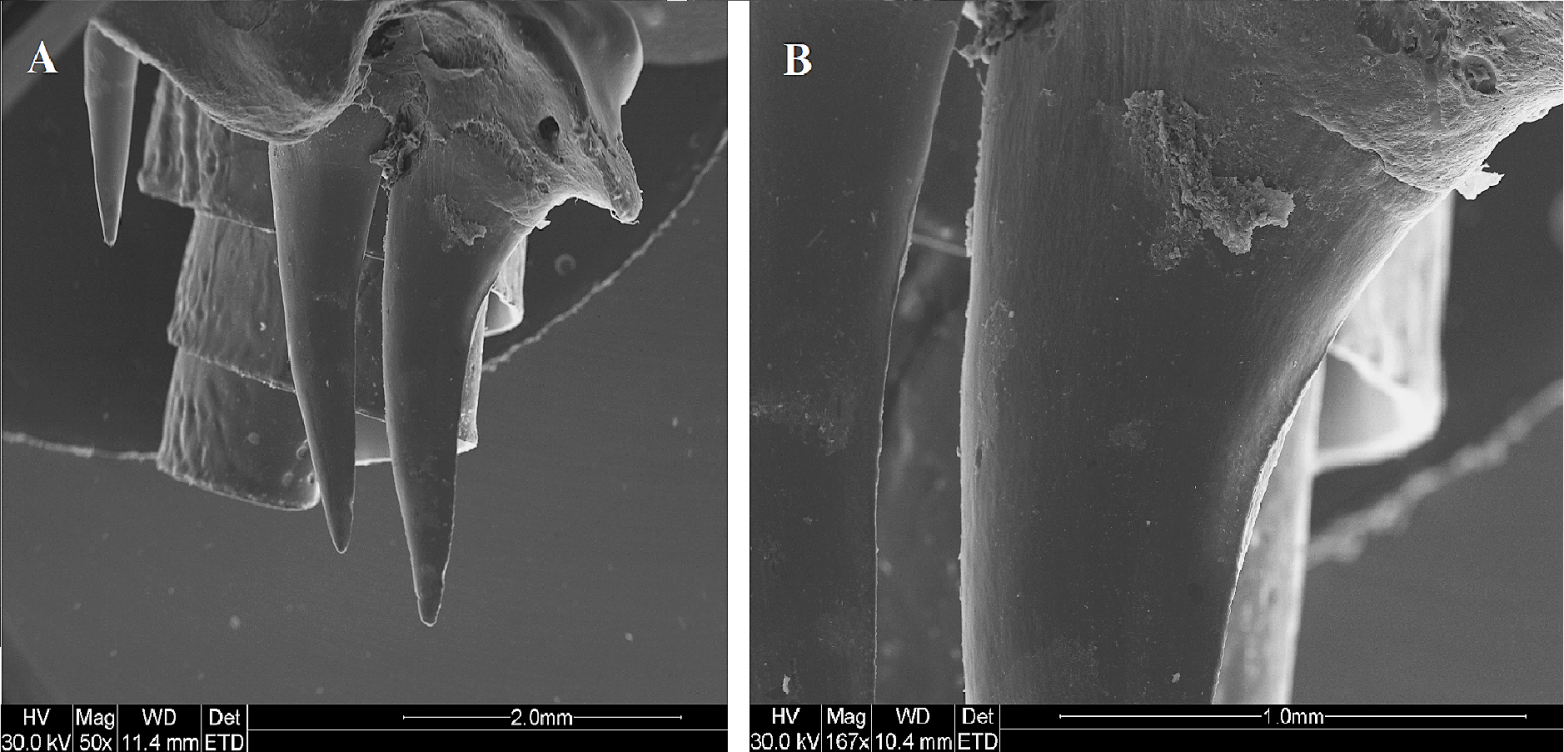
SEM (Scanning-Electron Microscopy) images of the posterior maxillary teeth (T12, T13) of a male mole snake (A) highlighting the blade-like carina along the posterior edge of T13 (B).

The maxillary teeth varied in size and shape along each maxilla bone, with noticeable differences between the anterior and posterior teeth (Fig. 2). The number of maxillary teeth was mostly consistent (13 teeth per side) across specimens, with one specimen in this study having 14 on one maxilla. The teeth at the anterior end and middle of the maxilla were long, conical, and recurved at the tooth base (Fig. 2). The two most posterior teeth (T12 and T13) were distinctive in shape, being labio-lingually flattened and triangular rather than conical and, in some specimens, pointed almost directly posteriorly from the tooth base, rather than perpendicular and recurving slightly posteriorly to the supporting maxilla, as was the case for the more anterior teeth (Figs. 2 and 5). T12 and T13 also had sharp posterior/dorsal edges in all specimens (Fig. 5).

**Figure 6:**
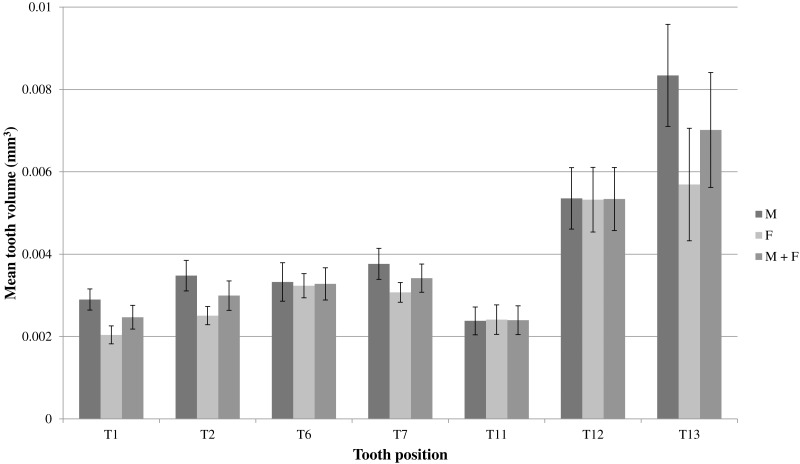
Mean volumes (mm^3^) and standard errors of front (T1 & T2), middle (T6 & T7) and back (T11–T13) maxillary teeth of seven male mole snakes (M) and seven female mole snakes (F).

An ANOVA test of the volumes of T1, T2, T6, T7, T11, T12 and T13 for the whole sample of mole snakes indicated that teeth located along the maxilla bone differed significantly in volume when compared to one other (*F*_(1,6)_ = 12.37, *p* <0.001; Table 1). A Tukey post-hoc test showed that the teeth at the posterior end of the maxilla (T12 and T13) were both significantly larger than T1, T2, T6, T7 and T11 (Table 1). The average T13 volume was more than double the volume of T1, T2, T6, T7 and T11 (*p* <0.01 for all comparisons) and T12 was a minimum of 40% larger than T1 (*p* <0.001), T2 (*p* <0.01) and T11(*p* <0.001) (Fig. 6; Table 1).

**Table 1:** Significant results of the ANOVA and *post hoc* tests comparing volumes of teeth at different positions on the maxilla for the whole sample (14) and for the separate sexes (7m, 7f) of mole snakes.

Hypothesis test result	Pairwise tooth comparison	Whole sample	Males	Females
ANOVA *p*-value		<0.000001	0.000029	0.000089
ANOVA F statistic		12.370000	7.153000	6.292000
Tukey *post hoc p*-value	T12-T1	0.000093		0.001468
	T13-T1	<0.000001	0.000568	0.001129
	T12-T11	0.000015	0.009328	0.005811
	T13-T11	0.000001	0.000023	0.004531
	T2-T12	0.009367		0.028693
	T2-T13	0.000042	0.006182	0.022934
	T6-T13	0.000447	0.002442	
	T7-T13	0.001529	0.017644	

### Sex-based differences in tooth shape and volume

The most pronounced sex difference in dentition was in the dentary teeth. All male specimens had posterior carinae (sharp edges) on 5 to 13 teeth on each dentary bone. Females lacked the sharp posterior edges on their dentary teeth, their teeth having rounded edges when compared to the distinctively sharp teeth in the males. Each specimen had between 14 and 16 teeth on each dentary bone, but the variation in tooth number was not sex-specific (Wilcoxon rank sum test, *W* = 27.5, *p* >0.9).

On average, teeth located along the maxilla bone differed significantly in volume when compared to one other, for both male and female samples (One-way ANOVAs for each sex, males: *F*_(1,6)_ = 7.153, *p* <0.001; females: *F*_(1,6)_ = 6.292, *p* <0.001; Table 1). A Tukey post-hoc test indicated that in males, the mean T13 was larger than T1 (*p* <0.001), T2 (*p* <0.01), T6 (*p* <0.01), T7 (*p* = 0.02) and T11 (*p* <0.001), and T12 was larger than T11 (*p* <0.01) (Table 1). In females, T13 was larger than T1 (*p* <0.01), T2 (*p* = 0.02) and T11 (*p* <0.01), and T12 was similarly larger than T1 (*p* <0.01), T2 (*p* = 0.03) and T11 (*p* <0.01) (Fig. 6; Table 1).

**Figure 7:**
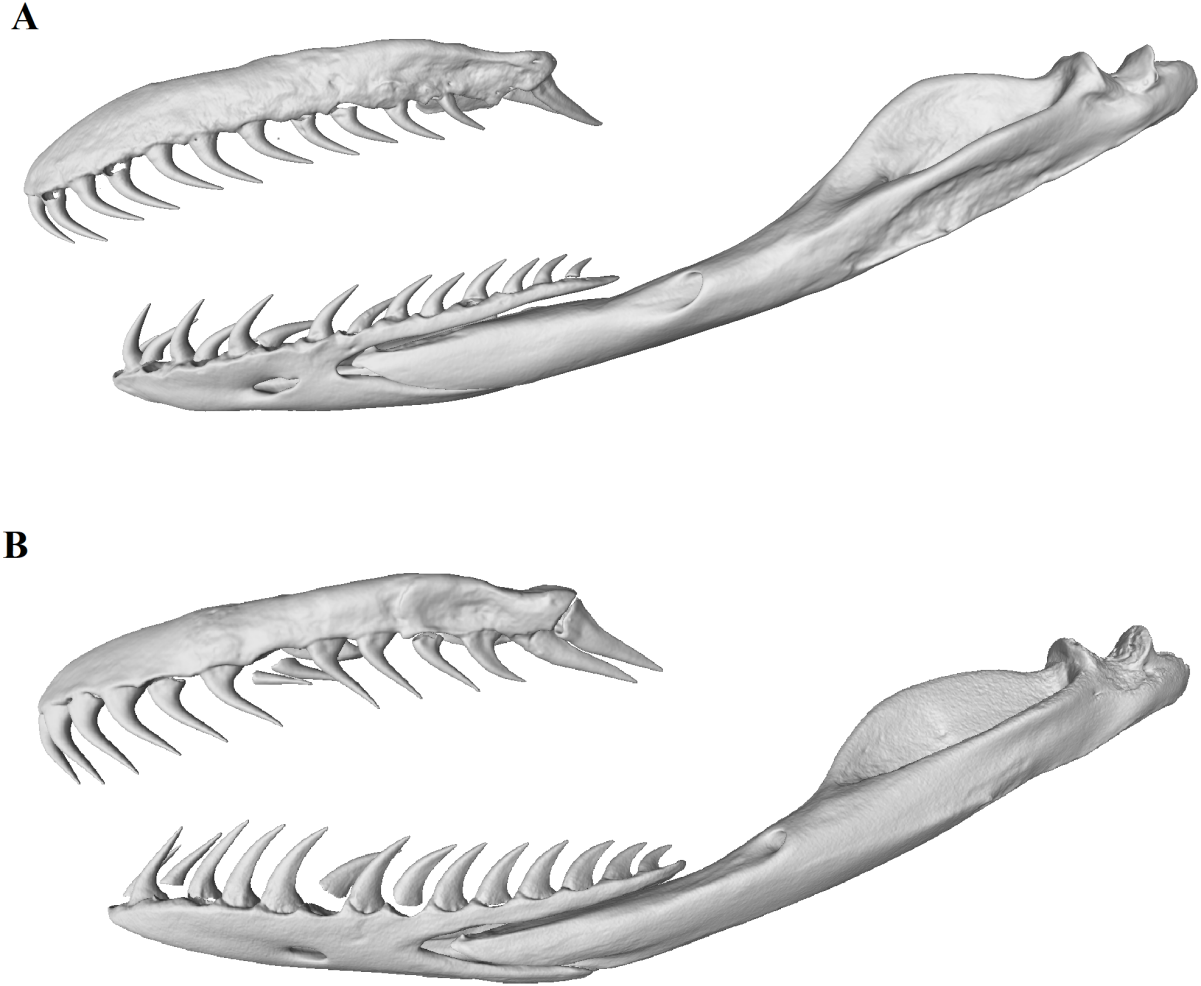
Micro-CT (computed tomography) reconstructions comparing the lateral view of the left maxilla, dentary and compound bones of a female (A) with that of a male (B) mole snake.

The distinct shapes and greater sizes of T12 and T13, compared to the other teeth, appeared more pronounced in males than in females (Figs. 6 and 7). A Wilcoxon rank sum test failed to detect a significant difference between the sexes for mean volume of T12 (*W* = 24, *p* > 0.9) and T13 (*W* = 36, *p* = 0.165). However, a multifactoral ANOVA with tooth number, sex and the interaction of sex and tooth as factors did reveal an effect of sex on tooth volume (*F*_(1,1)_ = 5.512, *p* = 0.0213), indicating that sex contributed to the differences in mean tooth volumes between individuals (seen in Fig. 6). There was no difference between the male and female mean lower jaw volumes (male 46.9 ± 6.3 mm^3^, female 44.5 ± 5.1 mm^3^; *W* = 23, *p* = 0.90) indicating that the differences in the relative maxilla tooth measurements were not artefacts of differences in jaw volumes.

### Scars on museum specimens

The proportion of male specimens with scars or wounds was significantly higher than the proportion of females with scars or wounds (*χ*^2^ = 5.5987, *d*.*f*. = 1, *p* = 0.018). Out of 50 museum specimens examined (28 adult male, 14 adult female and 8 juvenile), 9 adult males and 1 adult female showed signs of scars or wounds of various lengths and depths across the axis of the body. Six of these males had scars in the tail region, ranging from a scar spiralling twice around the tails on two specimens (such as in Fig. 1A) to a cut or scar over the ventral surface of the tail in three specimens, to a small, shallow scar across only part of the dorsal surface in one specimen. This specimen also had parallel gashes penetrating through the ventral scales and into the muscle towards the middle of the torso area. The scars of the remaining three males were found towards the middle of the snake and similarly varied in severity, from one long scar across the ventral surface of the body to multiple small scars not covering the width of the snake over both the dorsal and ventral surfaces of the body. The single female had a shallow scar above the tail that was half the width of the dorsal surface at that point.

## Discussion

The ability of the mole snake to inflict cut-like bites has been noted by a number of field guides and unwary handlers, but the dentition that causes this, and whether this dentition differs between the sexes, had not been reported before this study. A clear signal for sexual dimorphism is evident in the mole snake’s dentary teeth, a number of which have conspicuously sharp posterior edges (carinae) in male specimens only. In both sexes, the two posterior-most teeth on the maxilla are noticeably larger, labio-lingually flattened, and their apicobasal axis is strongly oriented posteriorly, whereas in more anterior maxillary teeth it is oriented dorsoventrally. Although these maxillary dental characters are present in both sexes, indicating a possible sex-nonspecific function, the posterior maxillary teeth are more pronounced in males. The sexual dimorphism in the teeth, as well as in the higher incidence of lacerations present on male specimens, indicates that male mole snakes have specialised dentition that aids in combat.

It is likely that the sharp dentary teeth as well as the posterior maxillary teeth play an important function in creating the cutting wounds typical of the mole snake’s bite. When the mouth is opened to its full extent, the posterior maxillary teeth (T12 and T13) point downwards and will likely be the first teeth to make contact with the target, as in some other snakes (Wright, Kardong & Bentley, 1979). Behavioural studies are required to ascertain the exact motion of the jaws and teeth during a bite, but we hypothesise that there are two ways in which the mole snake may bite to cause these wounds: the posterior maxillary teeth anchor the target while the lower jaw, aided by the double articulation of the quadrate bone, is levered backwards, allowing the dentary teeth to cut across the prey, a hypothesis that is consistent with the presence of more serious wounds on the ventral surfaces of the museum specimens, or the posterior maxillary teeth cause the wounds when the maxilla first makes contact with the rival snake during a bite.

Lacerations like those caused by mole snake bites have not been documented in many non-venomous snake species, with the only comparable species being the Australian carpet python (*Morelia spilota*) and the kukri snake (*Oligodon formosanus*), both of which have been observed participating in aggressive conspecific combat (Shine & Fitzgerald, 1995; Huang et al., 2011). Like the mole snake, the carpet python has been observed inflicting severe, gash-like bites on rival males when competing for a mate but the area of the body to which the bites are directed is not documented. The kukri snake’s enlarged maxillary teeth serve to inflict slashing wounds on rivals (sex-nonspecific) when defending feeding territory, and aid in slitting open turtle eggs (Huang et al., 2011). Male kukri snakes are more likely to incur bites to their tails than are females but the bites incurred by male kukri snakes tend to be less severe than those in females. Huang et al. (2011) propose that this evolved in response to the risk of damaging the reproductive organs.

In contrast to the kukri snake, male mole snakes are more likely to exhibit damage to their tails (and to some degree, torsos) than are females. The presence of posterior carinae on the dentary teeth of male mole snakes and the lack of this feature in females, as well as the more noticeably enlarged posterior maxillary teeth of the male mole snake suggest that these features may provide some advantage, such as during the male combat observed in the breeding season (Alexander & Marais, 2007; Broadley, 1983). From an adaptationist standpoint, the benefit of having teeth that cause long or spiral cuts in combat, rather than the more common puncture wounds, may be to inflict greater damage on the competing male’s tail, where the hemipenes are located, decreasing the rival’s chances of reproductive success in order to increase the number of females available for the victor. We note, however, that we cannot conclude that females do not inflict bites on males, and also that the tail may simply be the most accessible part of a male’s anatomy during combat (Huang et al., 2011). The chance of damaging the rival’s reproductive organs, even if not aiming for this region specifically, could have caused this behaviour to become selected for in the population.

The presence of specialised maxillary teeth in both sexes of mole snake suggests that these teeth either provide similar advantages to each sex, or are the result of constrained evolution in females (*sensu* Gould & Lewontin, 1979; Gould, 1993). Specialised maxillary teeth are fairly common in colubroids (Vaeth, Rossman & Shoop, 1985; Scanlon & Shine, 1988; Jackson & Fritts, 1995) and tend to be correlated with specialization in diet (Knox & Jackson, 2010). The most likely adaptationist explanation for the presence of sharp, triangular posterior maxillary teeth in both sexes of the mole snake is that these teeth are associated with special feeding requirements. Enlarged posterior maxillary teeth (and in some species, pterygoid teeth) are typically associated with the consumption of slippery prey such as fish (Wright, Kardong & Bentley, 1979) or oophagy (Scanlon & Shine, 1988). Although the diet of mole snakes is not well documented (adults appear to feed primarily on small burrowing mammals, and juveniles, primarily on lizards, Alexander & Marais, 2007; Broadley, 1983), the diet does not appear to be unusually specialized, nor does it consist of particularly slippery prey. However, Broadley (1983) and Dyer (1996) do report mole snakes consuming whole birds’ eggs, which may be an important component of the diet in some populations. Thus, the adaptationist diet hypothesis may be a partial explanation for the specialised maxillary teeth in mole snakes.

The sister taxon to the mole snake, the western keeled snake (*Pythonodipsas carinata*), has enlarged palatine ‘fangs’ that may aid in extracting prey from burrows (Branch et al., 1997) or for holding onto geckos (Marx et al., 1982). Similarly, hunting habitat rather than prey anatomy may have contributed to the selection for specialised teeth in the mole snake, if the teeth aided in dispatching prey in the restricted fossorial spaces in which mole snakes sometime hunt. However, there is little support in the literature for this and, in contrast to the mole snake, non-venomous fossorial snakes tend to have small, unvaried teeth (Savitzky, 1983).

An alternative explanation for the maxillary dental specialization in mole snakes is that the posterior teeth are phylogenetic remnants of ancestral fangs. Jackson (2007) argues that the ancestral condition in the Colubroidea was the presence of tubular fangs on the maxilla, and that nonvenomous species in this clade must therefore have subsequently lost fangs and the ability to produce venom. Furthermore she distinguishes ungrooved fangs from enlarged teeth by the presence of carinae along the rostral and caudal surfaces of the tooth—features that are present in the enlarged maxillary teeth in mole snakes. As per Kardong (1982), we do not define mole snake’s teeth as fangs, due to the absence of venom glands and the solely mucoid function of the Duvernoy’s glands (Taub, 1967). However, the presence of these teeth in mole snakes may indicate an origin of ancestral ungrooved fangs that have been co-opted into acting as an anchoring point, allowing the lower jaw to slice the body of male competitors, while being retained by proxy (through lack of selection to lose the teeth), or for the benefit in dispatching prey or consuming eggs, in females.

## Conclusions

Our observations shed light on the sparsely researched mole snake, and may serve as a basis for future functional studies on snake dentition, prey capture and sexual dimorphism. Further study could examine whether male mole snakes tend to bite rivals’ tails more than the rest of the body, and how tail damage affects reproductive success in mole snakes. Investigating hunting behaviour and skull kinetics may ascertain the exact mechanism of the mole snake’s bite, and functional comparisons with other species would contribute to a more comprehensive picture of the mole snake’s distinctive dental apparatus.

##  Supplemental Information

10.7717/peerj.6943/supp-1 Data S1 Volume measurements of the back, middle and front maxillary teeth, as well as the lower jaw, of each mole snake specimen

## Additional Information and Declarations

### Data Availability

The following information was supplied regarding data availability: The raw data is available at MorphoSource, Project: “The cutting-edge morphology of the mole snake’s dental apparatus”, https://www.morphosource.org/Detail/ProjectDetail/Show/project_id/633.
